# Scale dependency of ectomycorrhizal fungal community assembly processes in Mediterranean mixed forests

**DOI:** 10.1007/s00572-022-01083-4

**Published:** 2022-06-04

**Authors:** Prieto-Rubio J., Garrido J. L., Pérez-Izquierdo L., Alcántara J. M., Azcón-Aguilar C., López-García A., Rincón A.

**Affiliations:** 1 Department of Soil Microbiology and Symbiotic Systems, Estación Experimental del Zaidín (EEZ), CSIC, 1, Rd. Profesor Albareda, 18008 Granada, Spain; 2 Department of Soil, Plant and Environmental Quality, Instituto de Ciencias Agrarias (ICA), CSIC, Madrid, Spain; 3 Escuela Internacional de Doctorado, Universidad Rey Juan Carlos (URJC), Madrid, Spain; 4 Department of Evolutionary Ecology, Estación Biológica de Doñana (EBD), CSIC, Seville, Spain; 5 BC3 Basque Centre For Climate Change, Scientific Campus of the University of the Basque Country, Leioa, Spain; 6 Department of Animal Biology, Plant Biology and Ecology, Universidad de Jaén, Jaén, Spain; 7 Instituto Interuniversitario de Investigación del Sistema Tierra en Andalucía (IISTA), Granada, Spain

**Keywords:** Ectomycorrhizal fungi, Community structure, Assembly processes, Environmental filtering, Biotic interactions, Mediterranean mixed forests

## Abstract

The assembly of biological communities depends on deterministic and stochastic processes whose influence varies across spatial and temporal scales. Although ectomycorrhizal (ECM) fungi play a key role in forest ecosystems, our knowledge on ECM community assembly processes and their dependency on spatial scales is still scarce. We analysed the assembly processes operating on ECM fungal communities associated with *Cistus albidus* L. and *Quercus* spp. in Mediterranean mixed forests (Southern Spain), for which root tip ECM fungi were characterized by high-throughput sequencing. The relative contribution of deterministic and stochastic processes that govern the ECM fungal community assembly was inferred by using phylogenetic and compositional turnover descriptors across spatial scales. Our results revealed that stochastic processes had a significantly higher contribution than selection on root tip ECM fungal community assembly. The strength of selection decreased at the smallest scale and it was linked to the plant host identity and the environment. Dispersal limitation increased at finer scales, whilst drift showed the opposite pattern likely suggesting a main influence of priority effects on ECM fungal community assembly. This study highlights the potential of phylogeny to infer ECM fungal community responses and brings new insights into the ecological processes affecting the structure and dynamics of Mediterranean forests.

**Supplementary information:**

The online version contains supplementary material available at 10.1007/s00572-022-01083-4.

## Introduction

Understanding the ecological processes behind community organization is key to predict the establishment and coexistence of local species pools (Pearson et al. 2018). They have been classified into selection, dispersal, drift and speciation (Vellend 2010). Speciation operates at a wide temporal scale and it is expected to have little effect in organizing communities able to exchange individuals, i.e. belonging to the same metacommunity. The other processes can be classified into deterministic that may conduct selection from the regional species pool (e.g. species interactions and environmental filters), and stochastic, based on probabilistic events associated with population demography: drift (i.e. populations fluctuating by chance) and dispersal (i.e. the ability of individuals to move across communities) (Stegen et al. 2012; Ning et al. 2020). Dispersal may have contrasting effects: when it is limited, it enhances stochastic drift, whereas high dispersal may cause mass effects that homogenize communities (Stegen et al. 2013; Evans et al. 2017). Deterministic processes fit into the niche theory that assumes that species environmental requirements determine community assembly (Chesson 2000; Goberna et al. 2019; Viana and Chase 2019).

This theoretical scheme is dynamic and the relative importance of deterministic and stochastic processes varies across spatial and temporal scales (Chase and Myers 2011; Kivlin et al. 2014; Davison et al. 2016; Viana and Chase 2019). In fact, the effect of deterministic mechanisms such as environmental filtering is usually observed across broad abiotic gradients. Meanwhile, the importance of biotic interactions, together with stochastic processes, increases as the environment becomes more homogeneous at smaller scales (Götzenberger et al. 2012; Vályi et al. 2016).

Soil microbial communities show complex structural and functional responses to the environment that might hinder our understanding of community assembly processes (Pérez-Izquierdo et al. 2019). Microbial community features, such as phylogenetic relatedness (Miller et al. 2017) or functional traits (Martiny et al. 2013; López-García et al. 2018), are increasingly relevant in studying the assembly of microbial communities. Thus, to infer ecological processes, Stegen et al. (2013) proposed an analytical framework combining both the phylogenetic turnover (i.e. the averaged evolutionary distance among taxa in different communities) and compositional turnover of microbial communities. In fact, both abundance-based and phylogenetic information are currently steadily explored in microbial ecology by using null model approaches (Tripathi et al. 2018; Zhao et al. 2019; Pereira et al. 2020). Null modelling allows estimating the extent to which the structural patterns of the observed community differ from random expectations derived from stochastic processes (Chase and Myers, 2011; Münkemüller et al. 2020). This analytical approach may also link the dependency of assembly processes to spatial or temporal scales (Kivlin et al. 2014; Viana and Chase 2019; Zhao et al. 2019). Thus, Davison et al. (2016) demonstrated the shift from environmental filtering towards biotic interactions from broader to finer spatial scales by studying the phylogenetic structure of arbuscular mycorrhizal fungal communities collected worldwide. However, despite this potential, the null modelling approach has been barely implemented to analyse the scale dependency of ectomycorrhizal (ECM) fungal community assembly rules (but see Pickles et al. 2012).

The ECM symbiosis has key ecological and biogeochemical implications in forests, such as affecting net primary production and promoting nutrient mobilization through belowground mycelial networks (Lu and Hedin 2019; Rog et al. 2020; Tedersoo et al. 2020). The species composition of ECM fungal communities is influenced by geographical and abiotic environmental conditions (e.g. climate, seasonality or soil properties, Rincón et al. 2015; Pérez-Izquierdo et al. 2017; van der Linde et al. 2018), biotic factors such as the host partner (Pérez-Izquierdo et al. 2017; Wang et al. 2019), dispersal ability and fungal life-history differences across taxa (Peay et al. 2012) and the interaction of both biotic and abiotic factors (Buscardo et al. 2010; Põlme et al. 2013). However, the spatial hierarchy of assembly processes governing ECM fungal communities remains unclear (Zhao et al. 2019) and particularly in the Mediterranean area where such kinds of studies are scarce. The Mediterranean basin is a biodiversity hotspot where species have diversified by adapting to particular environmental conditions, such as large soil heterogeneity, severe seasonal droughts or fire recurrence (Rundel et al. 2016; Pérez-Valera et al. 2018; Pérez-Izquierdo et al. 2020). These adaptations have led to highly diverse biological communities with complex assembly processes (Rincón et al. 2014; Alcántara et al. 2018).

Under this environmental context, we aimed to identify the main potential factors and underlying processes explaining ECM fungal community structure in Mediterranean mixed forests. We also wanted to study the habitat scale dependency of these assembly processes. We expected that ECM fungal communities would be deterministically assembled due to the relatively high dispersal potential of this fungal group (Peay et al. 2012) (hypothesis 1). That notwithstanding, and as formerly stated (Stegen et al. 2013; Vellend et al. 2014; Peay 2018), the relative importance of deterministic (environmental filtering) vs. stochastic (dispersal limitation and drift) assembly processes should change with the spatial scale, with stochastic forces increasing in importance at smaller scales (hypothesis 2). To test these hypotheses, we analysed, at different spatial scales, the structure of root tip ECM fungal communities of three dominant plant species of Mediterranean mixed forests in Southern Spain.

## Materials and methods

### Study site and sampling design

**Fig. 1:**
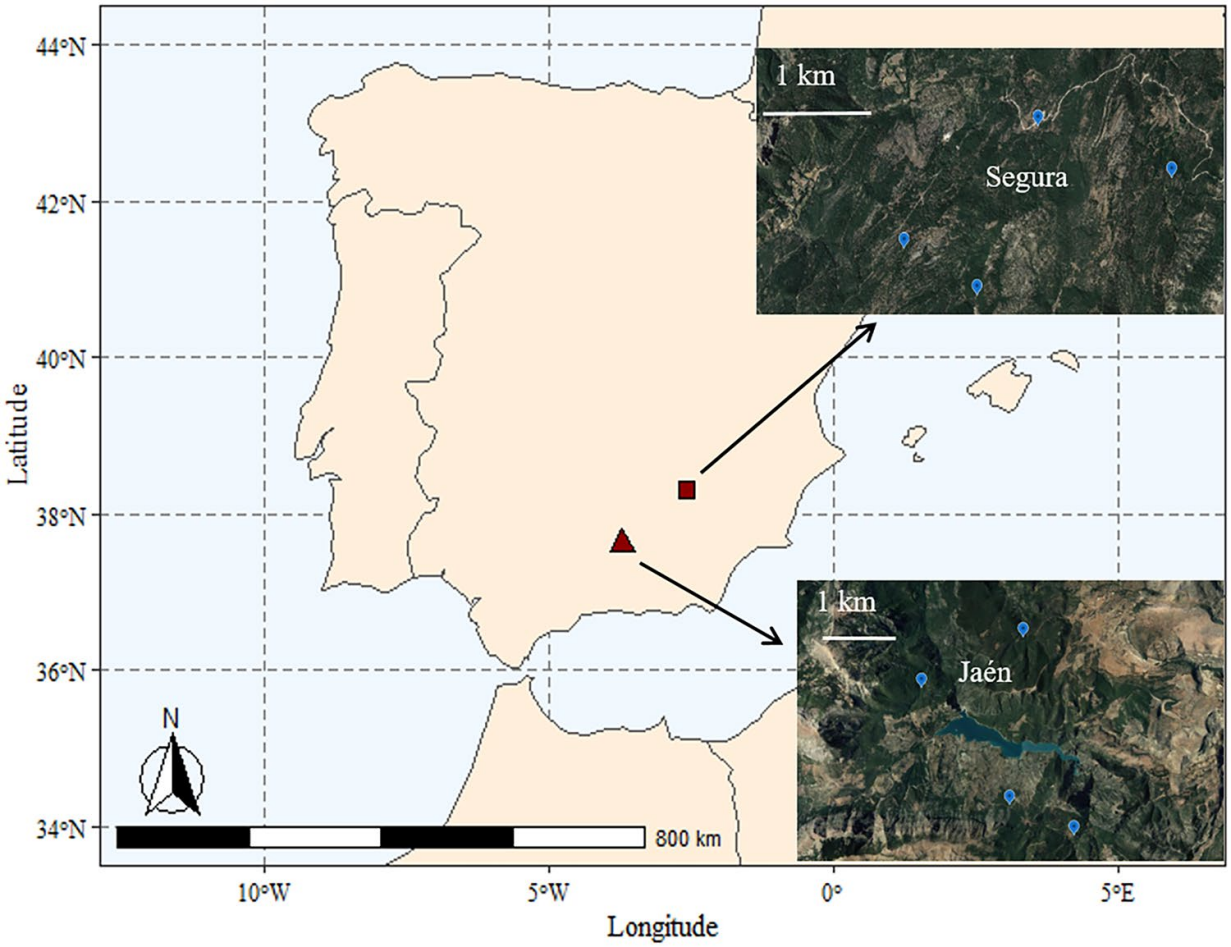
Location of the study sites and plots in Southern Spain: Sierra Sur de Jaén park (referred as Jaén, triangle), and Sierras de Cazorla, Segura y las Villas natural park (referred as Segura, square)

This study was conducted on mixed forests located in two protected areas in Southern Spain (hereafter sites) (Fig. 1): Natural Park of Sierras de Cazorla, Segura y Las Villas (38.29°N, − 2.57°W, hereafter called Segura) and Sierra Sur de Jaén Park (37.64°N, − 3.73°W, hereafter called Jaén). Mixed forests of *Pinus halepensis* Mill., *Quercus ilex* L. and *Quercus faginea* Lam. dominated in Jaén, and mixed forests of *Pinus nigra* subsp. salzamanii J.F. Arnold, *Pinus pinaster* Ait., *Q. ilex*, *Q. faginea* and *Quercus pyrenaica* Willd. in Segura (Pulgar et al. 2017; Alcántara et al. 2018). The understory plant species often found was highly diverse, belonging to the genera Cistus, Crataegus, Juniperus, Rosmarinus and Thymus, among others. Both sites show similar geological context (Siles et al. 2010). In the region, the climate is continental Mediterranean, with an annual mean rainfall of 800–1000 mm and a mean annual temperature of 10–12 °C.

Experimental design and sample processing and analyses are detailed in Suppl. Info. Appendix 2. Briefly, three plant species were sampled at both sites (*Cistus albidus* L., *Q. faginea* and *Q. ilex*). We collected ECM root tips from four individuals per plant species in four plots per site that were molecularly analysed (final *n* = 92) by Illumina Miseq (ITS-1 rDNA region). Gravimetric moisture (GM), pH and organic matter (OM) content of soils were also determined. Raw sequences were processed with DADA2 pipeline v1.16 (Callahan et al. 2016; R Core Team 2021) and the LULU algorithm (Frøslev et al. 2017). Operational taxonomic units were obtained by clustering amplicon sequence variants at a 97% similarity and their taxonomy was checked against UNITE database v7.2 (Abarenkov et al. 2010). OTUs were classified by fungal guilds with the FUNGuild database v1.0 (Nguyen et al. 2016). The final output yielded 449 OTUs and 6,582,941 reads related to the ECM lifestyle. Homogeneous sequencing depth across samples was confirmed by rarefaction (vegan R package, Oksanen et al. 2019) (Fig. S1).

### Plant and ECM fungal phylogenies

The phylogeny of plant species used in this study has been previously published by Alcántara et al. (2018). The ECM fungal community phylogeny was estimated with the program Phylomatic as implemented in Phylocom 4.2 (Webb et al. 2008) and the BEAST software v.1.10.4 (Suchard et al. 2018). The phylogenetic tree was generated with a reference fungal mega-tree, whose topology and age for major nodes were inferred from the phylogenetic information available in the literature (Pérez-Izquierdo et al. 2019). The input for Phylomatic was the list of fungal OTUs that was matched to the most resolved position in the mega-tree previously constructed, so that if any genus was missing from the mega-tree, a polytomy of genera within that family was returned (Moles et al. 2005). For the remaining undated nodes, ages were estimated with the BLADJ algorithm in Phylocom (Webb et al. 2008) that distributes undated nodes evenly between nodes of known ages. To check for the robustness of results to the topological and chronological uncertainty introduced by the Phylomatic and BLADJ procedures, an additional phylogenetic tree based on a branch length adjustment procedure that follows a birth–death evolutionary model while randomly resolves the polytomies in the BEAST program was constructed (Drummond and Rambaut 2007; Kuhn et al. 2011). Markov Chain Monte Carlo (MCMC) analyses for 5 × 10^6^ iterations were run, sampling trees every 10^3^ iterations, discarded a 25% burn-in and recovered the maximum clade credibility tree using the TreeAnnotator v1.5.4 software (Drummond and Rambaut 2007).

### Data analyses

#### Drivers of ECM fungal community composition

The distribution of measured soil variables (pH, OM and gravimetric soil moisture previously log transformed) was tested via linear mixed-effect models (LMMs) with host plant species identity and season as fixed factors, and site and plot nested in site as random factors. The interaction host plant × season was not significant and it was discarded from models. The same model was used to analyse the nearest taxon index (NTI) (Webb et al. 2002) as a measure of phylogenetic distance between OTUs in a single community. NTI allows the inference of potential assembly processes, i.e. phylogenetic similarity or clustering of fungi would indicate the action of an environmental filter, while overdispersion that of competition. NTI was quantified with the ses.mntd function and taxa.labels as null model (999 null communities in picante R package) and the relative abundance and the phylogenetic distance matrices of the ECM fungal communities. The significance of fixed and random factors was determined with the Anova (car R package) and rand functions (lmerTest R package) respectively, and their relative contribution by the coefficient of determination (pseudo-*R*^2^) calculated with the r.squaredGLMM function (MuMIn R package). Significant differences of predicted means were separated by the Tukey test (HSD.test function, agricolae R package).

Phylogenetic relatedness is a good proxy for shared traits of species, allowing to identify environmental filters driving community assembly (Martiny et al. 2015; Goberna and Verdú 2016; Rog et al. 2020). Thus, we used the β-nearest taxon index (βNTI), which is the between-assemblage analogue of NTI (Fine and Kembel 2011). βNTI was calculated with qpen function and taxa shuffle as a null model (999 null communities in iCAMP R package).

To disentangle the weight of biotic (plant species), abiotic (environmental properties) factors, and stochastic events in driving phylogenetic turnover (βNTI) of the fungal community, we subjected to principal component analysis (PCA) the soil abiotic variables, the spatial variables—previously decomposed via principal coordinates of neighbourhood matrix (PCNM, Borcard and Legendre 2002)—and the two principal coordinates axes of phylogenetic distances among plant species (Stegen et al. 2013). The significant influence of PCA axes on βNTI was tested by forward selection based on distance-based redundancy analysis (dbRDA) (ordistep and capscale functions in vegan R package).

To determine if any environmental or spatial variable could be driving species composition independently of their phylogeny, we applied the approach described for βNTI to the Raup Crick metric based on Bray–Curtis dissimilarity (RCBray) that measures the turnover of OTUs composition against that expected by chance (Stegen et al. 2013). This metric was calculated with the qpen function and taxa shuffle as the null model (999 null communities in the iCAMP R package).

Finally, to check the fungal taxonomic groups related to the community phylogenetic turnover, we calculated the principal coordinate axes (PCoA) of βNTI (function pcoa in the ape R package). PCoA axes were correlated with the significant PCA axes, derived from the forward selection, by applying the Spearman method with Bonferroni adjustment, using the corr.test function in the psych R package. PCoAs significantly related to PCA axes were tested against the relative abundance of main fungal phyla (Ascomycetes, Basidiomycetes) and families, following the same correlation analysis procedure as described above.

The main trends of ECM fungal community composition were represented by using a non-metric multidimensional scaling (nMDS) (Bray–Curtis dissimilarity, metaMDS function, vegan R package), where host plant species and site were plotted together with the abundance of ECM fungal taxa (envfit function 999 permutations, vegan R package).

#### Ecological assembly processes acting on ECM fungal communities across spatial scales

**Fig. 2:**
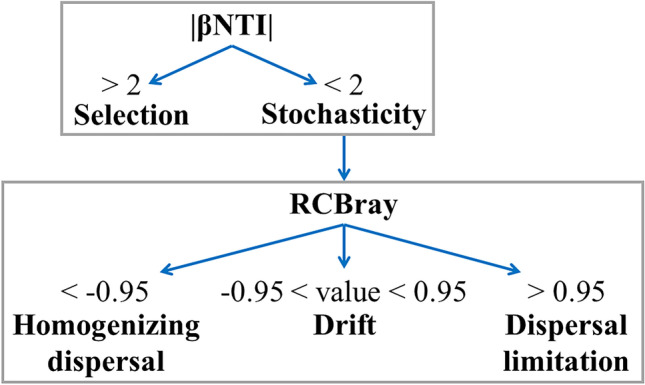
Schematic workflow for the inference of deterministic and stochastic processes through the community phylogenetic (βNTI, expressed in absolute values) and compositional (RCBray) turnover indices

To estimate the relative importance of the different assembly processes operating in the ECM fungal communities (hypothesis 2), we followed the analytical approach described by Stegen et al. (2012, 2013) (Fig. 2). We first discriminate among deterministic (i.e. selection) and stochastic processes by the βNTI values calculated for each pair of samples. When |βNTI|> 2, i.e. when the phylogenetic turnover is significantly smaller (βNTI < − 2) or greater (βNTI > 2) than expected by chance, it indicates that community assembly is affected by habitat filtering processes (selection). When |βNTI|< 2 (i.e. − 2 < βNTI < + 2), βNTI values are non-different from random expectations showing communities mainly driven by dispersal and/or drift processes (stochasticity) (sensu Stegen et al. 2013). To more finely delimit the stochastic processes, the matrix of RCBray values was calculated with the qpen function in iCAMP R package. RCBray > 0.95 that did not show |βNTI|> 2 assigned dispersal limitation processes operating in community assembly, RCBray < − 0.95 that did not show |βNTI|> 2 indicated homogenizing dispersal (i.e. high dispersal ability of ECM fungal species) and |RCBray|< 0.95 assigned drift as the determinant of community assembly (Fig. 2, Stegen et al. 2013). Both metrics (βNTI and RCBray) were calculated across spatial/habitat scales (regional, site, plot nested in site and host plant species nested in plot and site) by restricting the input abundance matrices, used for calculating the null communities, to regional, site, plot and host plant species, respectively. The relative contribution of each assembly process at each scale was determined as the number of community-community turnover assigned to each assembly process. A *χ*^2^ test was used to detect significant differences in the proportion of assembly processes across spatial scales.

Finally, to seek for the presence of species interactions driving the assembly of ECM fungal communities across scales, NTI values were calculated for each spatial scale, similar to what was done with the previous metrics. The significance of averaged NTI values at each scale respect to the null expectation was assessed by means of *t* test (*p* < 0.05) and differences between spatial scales were evaluated by the Tukey test with the function HSD.test in the agricolae R package.

All analyses conducted in this study were carried out with functions and packages of the R free software v.4.1.1 (R Core Team 2021).

## Results

The studied sites (Fig. 1) differed in soil properties, with higher pH and OM found in Jaén (pH 7.7 ± 0.1 and OM 16.5 ± 2.1%) than Segura (pH 6.6 ± 0.2 and OM 8.9 ± 1.0%) (Tables 1 and S1). Soil pH varied significantly between host plant species, but differences were mainly driven by samples from Segura, where *C. albidus* (7.1 ± 0.2) showed higher values than *Quercus* spp. (6.4 ± 0.1; 6.3 ± 0.2).

**Table 1:** Effect of the fixed factors season and host plant identity and the random factors site and plot (nested in site) on soil parameters and ectomycorrhizal fungal diversity indices, analysed by linear mixed-effect models. *F* and *χ*^2^ values with sub-indexes indicating the degrees of freedom for each factor are respectively given for fixed and random factors, and significant effects are noted in bold. The coefficient of determination (pseudo-*R*.^2^, i.e., variance explained) is shown for fixed and random factor pools

	**Fixed factors**			**Random factors**		**Pseudo-*R***^2^	
	**Season**		**Host plant identity**	**Site**	**Plot (site)**	**Fixed**	**Random**
*Soil parameters*							
pH	***F***^**2**^_**1**_ = 25.44 ***		***F***^**2**^_**2**_ = 5.40 **	***χ***^**2**^_**1**_ = 13.56 **	*χ*^2^_3_ = 1.11 ns	0.09	0.70
OM	*F*^2^_1_ = 0.92 ns		*F*^2^_2_ = 0.99 ns	***χ***^**2**^_**1**_** = 3.15**	***χ***^**2**^_**3**_ = 13.09 ***	0.01	0.58
GM	***F***^**2**^_**1**_ = 56.47 ***		*F*^2^_2_ = 2.06 ns	*χ*^2^_1_ = 0.00 ns	***χ***^**2**^_**3**_ = 14.03 ***	0.32	0.19
*ECM community diversity*							
S_Total_		*F*^2^_1_ = 2.35 ns	***F***^**2**^_**2**_** = 2.43**	*χ*^2^_1_ = 0.45 ns	***χ***^**2**^_**3**_** = 3.82**	0.06	0.17
S_Ascomycetes_		*F*^2^_1_ = 0.11 ns	***F***^**2**^_**2**_ = 9.40 ***	*χ*^2^_1_ = 0.41 ns	***χ***^**2**^_**3**_** = 2.71**	0.16	0.14
S_Basidiomycetes_		***F***^**2**^_**1**_ = 4.51 *	*F*^2^_2_ = 0.57 ns	*χ*^2^_1_ = 2.06 ns	*χ*^2^_3_ = 2.66 ns	0.04	0.26
NTI		***F***^**2**^_**1**_ = 4.37 *	*F*^2^_2_ = 1.11 ns	*χ*^2^_1_ = 1.43 ns	*χ*^2^_3_ = 0.00 ns	0.07	0.05

*ns* non-significant, *OM* organic matter (log transformed), *GM* gravimetric moisture (log transformed), *S* fungal richness (log transformed), *NTI* nearest taxon index calculated for the full system

***p < 0.001

**p < 0.01

*p < 0.05

. *p* < 0.10

**Fig. 3:**
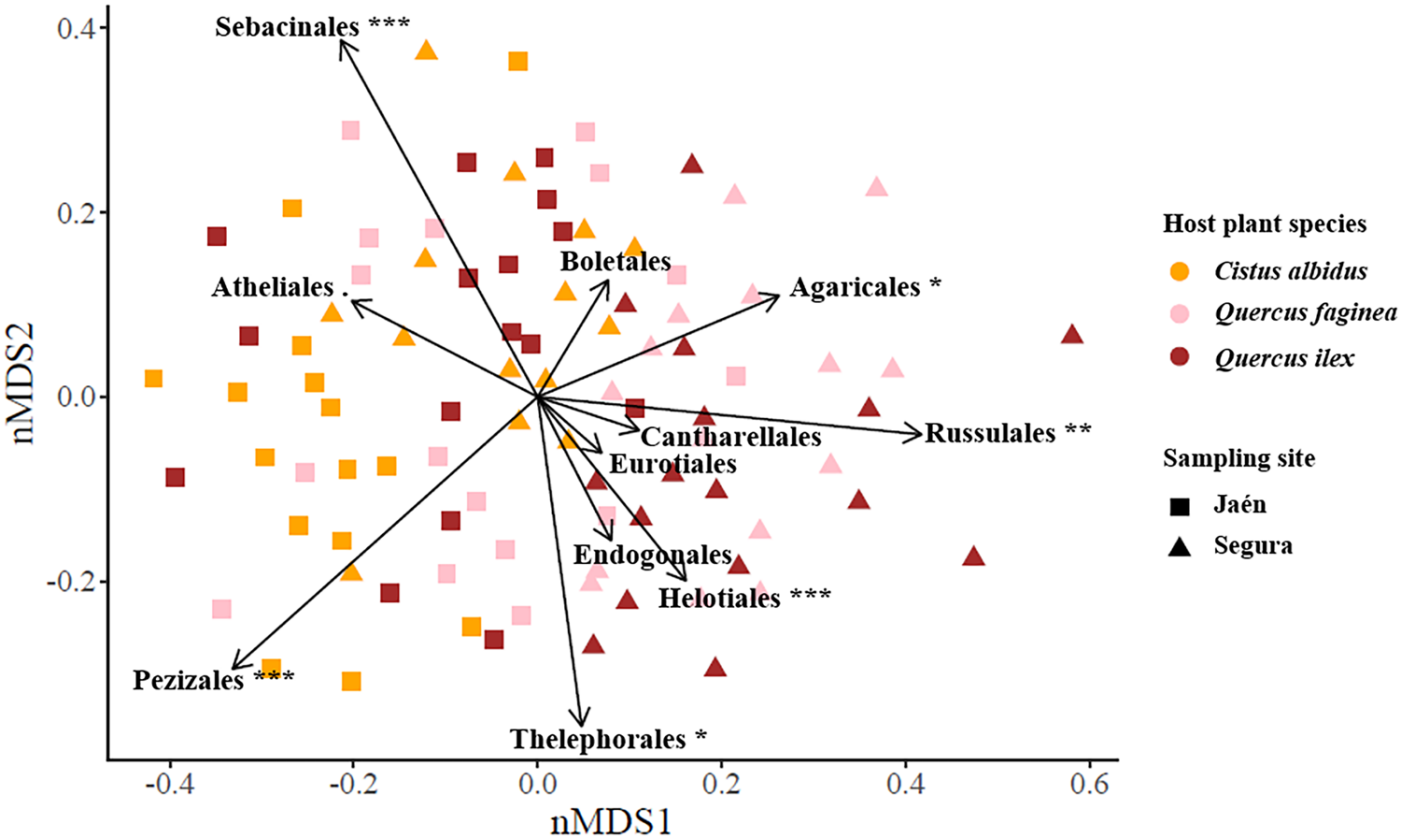
Structure of ectomycorrhizal fungal communities associated with the plant species of this study: *Cistus albidus*, yellow; *Quercus faginea*, pink; and *Quercus ilex*, brown. Fungal community composition was analysed by non-multidimensional scaling (stress = 0.29) on the Bray–Curtis dissimilarity matrix. Strength and direction of vectors indicate the relative weight of occurring fungal orders in structuring ECM fungal communities (correlation significance: ‘***’ *p* < 0.005, ‘**’ *p* < 0.01, ‘*’ *p* < 0.05, ‘.’ *p* < 0.1)

The ECM fungal community dissimilarity did also vary across sampling sites and host plant species (Tables 1 and S1; Fig. 3). The nMDS revealed that the abundance of Pezizales significantly correlated with *C. albidus* in Jaén and that abundance of Russulales and Helotiales did correlate with *Quercus* spp. in Segura (Fig. 3). NTI values were positive at all spatial scales analysed, and different from null expectations (Tables S1 and S2), pointing to phylogenetic clustering of ECM fungal communities.

### Drivers of ECM fungal community composition

**Fig. 4:**
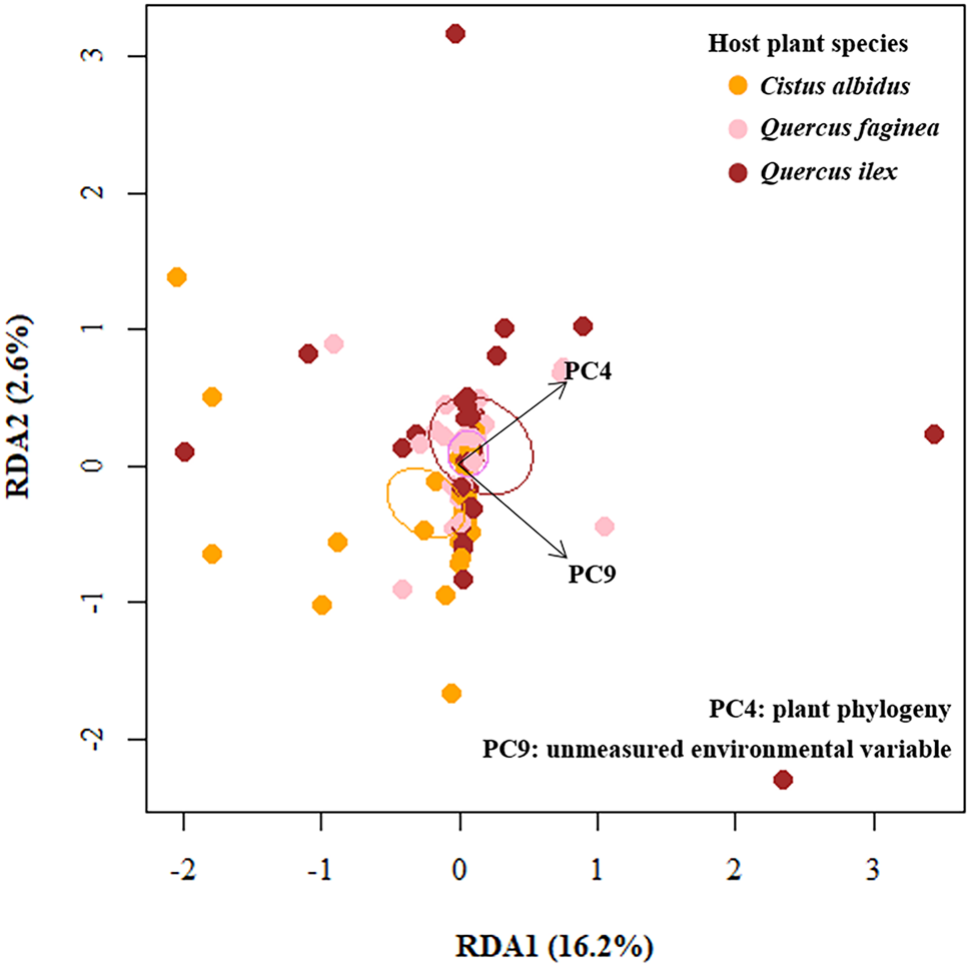
Effects of environmental variables on the phylogenetic turnover (βNTI) of ectomycorrhizal fungal communities. Distance-based redundant analysis (*F*_*2,89*_ = 3.37, *p* < 0.01) using significant principal components (PC) after forward selection (arrows). PCA 4 significantly correlated with the phylogenetic distance between *C. albidus* (yellow) and *Quercus* spp. (*Q. faginea* in pink and *Q. ilex* in brown) (see Table S3). Ellipses enclose βNTI values for each plant species (plotted by using the standard errors with *ordiellipse* function, *vegan* R package). PCA 9 significantly correlated with the narrowest decomposed spatial variable, indicating a spatially structured unmeasured environmental variable affecting ectomycorrhizal fungal phylogenetic turnover βNTI

Two PCA axes, composed by environmental and spatial variables, were found to drive the phylogenetic composition of ECM fungal communities (βNTI) (Fig. 4; Table 2). Depending of the components of significant PCA axes, they were interpreted as follows: those correlated to soil or plant phylogenetic variables were considered as ‘measured’ environmental filters, while PCA axes correlating with PCNM axes (spatial variables), but not with measured variables, were considered ‘unmeasured’ environmental filters (Table S3). Our results showed that PCA 4 mainly correlated with plant phylogeny (first PCoA axis, differentiating *C. albidus* from *Quercus* spp.; see Table S3), hence representing a ‘measured’ environmental filter. PCA 9 did also significantly correlate with βNTI, mainly due to PCNM 6, which represents the narrowest spatial scale and a spatially structured ‘unmeasured’ environmental filter.

**Table 2:** Forward selection based on distance-based redundant analysis (dbRDA) models of principal component analysis (PCA) axes (see Table S3) explaining phylogenetic (βNTI) and compositional (RC_Bray_) turnover of the ectomycorrhizal fungal communities. Depending on which variables loaded a PCA axis, it was classed as environmental measured variables (*ENV measured*) when heavier loadings were associated with soil variables and PCoAs (i.e. host plant) and spatially structured environmental unmeasured variables when spatial variables explained βNTI in the dbRDA (*ENV unmeasured*) (see Table S3)

**(A) Forward selection of variables driving βNTI**					
	**Adjusted *R***^**2**^	**Df**	**AIC**	***F***	***p***
PCA 9 *(ENV unmeasured)*	0.20304	1	89.991	3.3863	0.009**
PCA 4 *(ENV measured)*	0.39767	1	88.678	3.2636	0.010**
All PCA	1.0201				
**(B) Forward selection of variables driving RC**_**Bray**_					
		**Df**	**AIC**	***F***	***p***
PCA 3*(ENV measured)*		1	373.73	1.2638	0.099

*Df* degrees of freedom, *AIC* Akaike information criterion

*p* codes: ** < 0.01; . < 0.1

βNTI was decomposed into PCoA axes, and those axes significantly correlating with PCA 4 and 9 (Table S4) revealed the variation of certain fungal families. In fact, PCoA 5 and PCoA 36 (related to PCA 4, *r* = − 0.34, *p* < 0.001 and *r* = − 0.24, *p* = 0.02), correlated with the relative abundance of Gomphidiaceae plus Russulaceae (negatively), and Tuberaceae (positively), respectively (Table S4). By contrast, PCoA 9 (related to PCA 9, *r* = − 0.21, *p* = 0.04) did not correlate with any fungal family. In the case of PCoA axes 44 and 57 (related to PCA 9, *r* = 0.21, *p* = 0.05, and *r* = − 0.23, *p* = 0.03, respectively), only PCoA 57 significantly correlated with the abundance of Helvellaceae (positively) and Pezizaceae (negatively) (Table S4).

Focusing on compositional ECM fungal community turnover (RC_Bray_), whether a PCA axis did significantly drive it, but not that of βNTI, it was interpreted as an indication of dispersal limitation (i.e., limiting species distribution) or, alternatively, as an environmental driver acting on traits non-phylogenetically conserved. The RC_Bray_ outcomes were only marginally influenced by PCA 3, which was related to the second PCoA axis of plant phylogeny, i.e. differentiating equally between the three plant species (Table 2).

### Scale dependency of ECM fungal community assembly processes

**Fig. 5:**
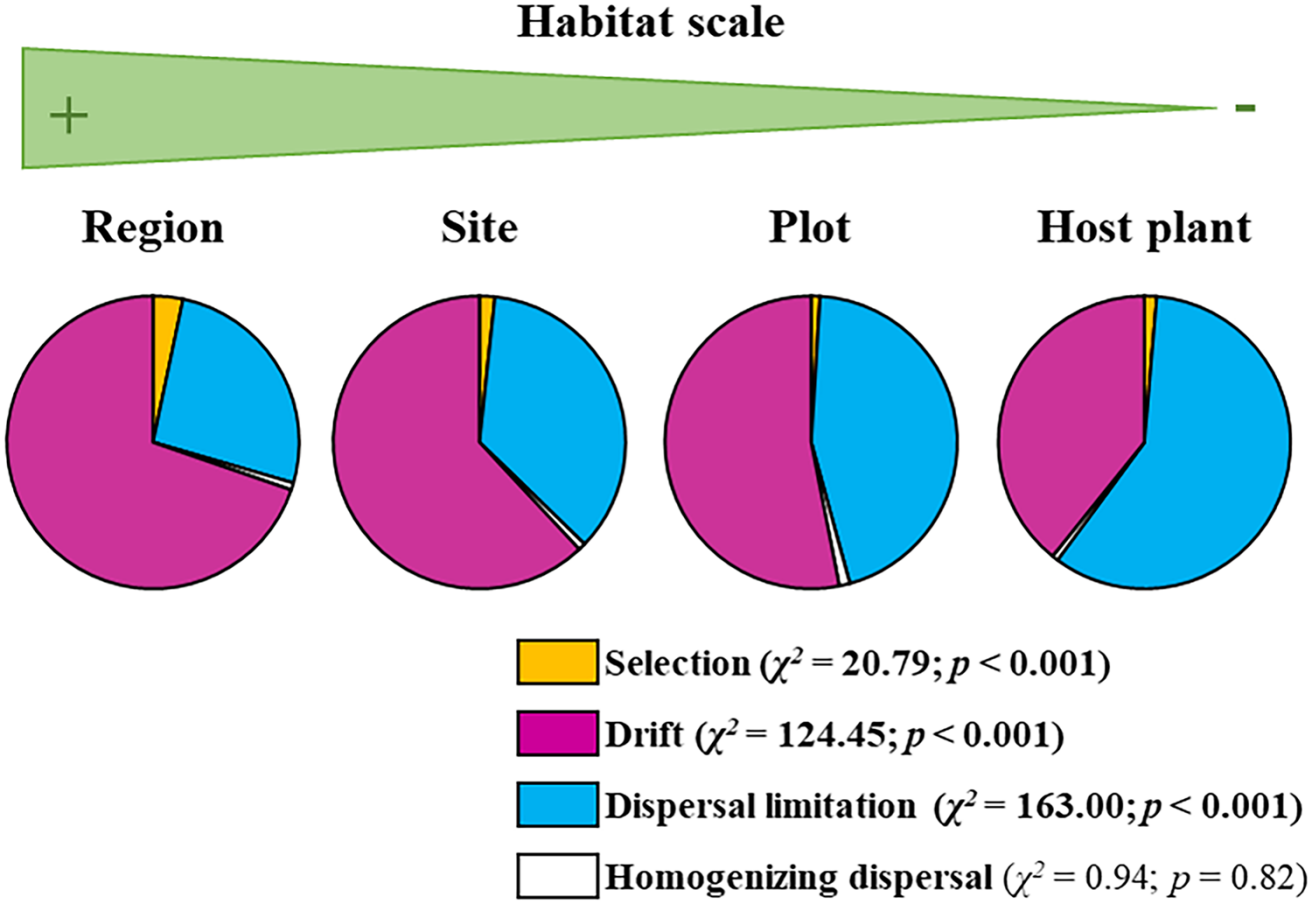
Percentage of ectomycorrhizal fungal community turnover explained by different assembly processes across habitat scales (region, site, plot nested in site and host plant species nested in plot and site). Differences across scales for each assembly process were assessed via a *χ*.^2^ test. Significant results are highlighted in bold (*p* < 0.05)

ECM fungal community turnover was explained to a different extent by different assembly processes, depending on the habitat scale (Fig. 5). Homogenizing dispersal was the process explaining the least number of community turnovers and it did not change with scale (*χ*^2^ = 0.94, *p* = 0.82). By contrast, selection (*χ*^2^ = 20.79; *p* < 0.001), dispersal limitation (*χ*^2^ = 163.00; *p* < 0.001) and drift (*χ*^2^ = 124.45; *p* < 0.001) did show significant variations across habitat scales. Selection explained a small percentage of community turnover and its importance decreased between the regional and the rest of the scales (Fig. 5). Drift decreased in importance when reducing the scale, while dispersal limitation followed the opposite pattern, increasing in importance when reducing the scale (Fig. 4).

Despite the general significant phylogenetic clustering revealed by the NTI analysis at every analysed habitat scale, it showed no variation across habitat scales (Table S2).

## Discussion

### Drivers of ECM fungal community composition

The fact that phylogenetic clustering was predominant across habitat scales indicates that environmental filtering is a widely distributed assembly force of root tip ECM fungal communities of the Mediterranean mixed forests, as previously reported in other forest ecosystems (Koide et al. 2011; Pena et al. 2017). Contrary to our expectations, we did not observe strong relationships between ECM fungal communities and the studied soil variables. If any, they were probably masked by the great effect of host plant identity (e.g. pH was significantly higher under *C. albidus* than *Quercus* spp.). The pH gradient, mediated by the plant species identity, could be a determinant to filter the regional ECM fungal species pool (i.e. Ascomycetes are usually tied to higher soil pH), as other studies have evidenced in soil microbial communities (Vályi et al. 2016; Glassman et al. 2017; Tripathi et al. 2018), and would explain the correlation of this fungal phylum with *C. albidus* (particularly Pezizales and the family Tuberaceae within this fungal order). Contrary, Gomphidiaceae and Russulaceae (Basidiomycetes) showed the opposite trend. Although the three soil variables analysed are commonly reported as the main environmental predictors of ECM fungal community assembly (Glassman et al. 2017; van der Linde et al. 2018), the phylogenetic turnover observed in our study system revealed that other non-measured spatially structured environmental variables (e.g. temperature, soil texture, quality of organic matter or soil key nutrients) might also explain ECM fungal community outcomes (Pena et al. 2017; van der Linde et al. 2018). This phylogenetic preference points towards the conservation of ecological traits in the evolution of ECM fungi (e.g. production of fruit bodies and spores, dispersal strategies, host preference or mycelium exploration type) (Treseder and Lennon 2015; van der Heijden et al. 2015; van der Linde et al. 2018). These results partially confirmed our first hypothesis: the ECM fungal community significantly depended on the host plant species identity and soil properties.

Despite the significant effect of environmental predictors, our results further revealed that all types of assembly processes described in Vellend (2010) had a role in configuring the ECM fungal communities. The magnitude of the influence of each process did largely vary. In fact, dispersal limitation and drift had a much greater role than deterministic (i.e. selection) processes contrary to our expectations (first hypothesis). This result suggests that stochastic mechanisms, such as priority effects (i.e. arrival timing of species into the community), could be key to understanding ECM community assembly outcomes (Peay 2018). Indeed, it is known that the early arrival of ECM fungal species to colonize root tips can limit the establishment of later arriving taxa, what would explain the role of dispersal limitation in the metacommunity (Kennedy et al. 2009; Napoli et al. 2010; Thoen et al. 2019). However, the lower extent of selection structuring ECM fungal communities could be an artefact of the used methodological approach. This approach relies on the assumption of phylogenetic conservatism of functional traits that, in the case of ECM fungi, could be challenged by their paraphyletic nature (Tedersoo et al. 2010). In any case, the fact that community compositional turnover (measured through RC_BRAY_) did not reveal any contribution of selection would also confirm the greater role of stochasticity over the selection on the ECM community assembly in our study system.

### ECM fungal community assembly processes were habitat scale dependent

The influence of assembly processes revealed differences with the analysed scale confirming our second hypothesis. Drift and dispersal limitation did explain most of this variation and both processes showed opposite patterns, being the former more influent at lower scale (host plant) and the last at higher scale (regional). Our results were consistent with the framework proposed by Zobel (1997) and previously tested by Davison et al. (2016) on arbuscular mycorrhizal fungal communities: the higher importance of dispersal limitation at finer scales, which might be related to the potential main role of priority effects also driven by competition, i.e. first taxa arriving within the system determine the establishment of the later taxa (Pickles et al. 2012). On the other hand, homogenizing dispersal showed a general low contribution to assembly and did not vary across habitat scales, suggesting that communities were not assembled by few highly abundant or highly dispersed taxa (i.e. mass effect) (Evans et al. 2017). As expected, selection generally diminished with habitat scales (Chase 2014; Zhao et al. 2019), likely influenced by those dominant ECM fungal taxa whose relatively high abundance could be reinforced by priority effects (Moeller and Peay 2016). The lack of differences in phylogenetic clustering across habitat scales seems to point out towards a lack of hierarchical effect of spatial scale on biotic interactions governing ECM fungal community assembly that has been previously described for other microbial groups (Götzenberger et al. 2012; Davison et al. 2016; Vályi et al. 2016; Goberna et al. 2019). In any case, the eventual absence of spatial hierarchical assembly might be supported by the functional redundancy of distantly related ECM fungal taxa (i.e. lack of phylogenetic conservatism on functional traits) (Pena et al. 2017). This is particularly important in environments such as the Mediterranean, where inter- and intra-annual environmental variability can lead to increasing stochastic events that would mask hierarchical assembly outcomes within communities. This idea would argue the need to complement the phylogenetic information obtained from the ECM fungal communities studied with other aspects of functional diversity (e.g. fungal traits, Põlme et al. 2021) in order to reveal the mechanisms underlying ECM fungal community assembly in Mediterranean forest ecosystems.

## Conclusions

In this study, we have found that (1) spatial factors and host plant species are determinants of ECM fungal community assembly in Mediterranean mixed forests; (2) phylogenetic and compositional turnover are good community structure proxies to evaluate the contribution of deterministic and stochastic assembly processes in ECM fungal communities and (3) the contribution of assembly processes is habitat scale dependent. Thus, ECM community assembly in Mediterranean forests is dependent on both deterministic and stochastic processes, particularly dispersal limitation and drift, increasing the first and diminishing the second at smaller scales. These patterns confirm the habitat scale dependency of assembly processes, as previous studies described in other biological guilds but not in ECM fungal communities. Further studies are needed to disentangle the role of fine-tuned biotic interactions in forests, as well as how ECM phylogeny complements with functional diversity and environmental drivers, particularly in Mediterranean ecosystems. Additional information may be found in the online version of this article at the publisher’s website.

## Supplementary information

Below is the link to the electronic supplementary material.

Additional information may be found in the online version of this article at the publisher’s website:

**Appendix 1**. Supporting information with figures and tables (DOCX 118 KB)

**Appendix 2**. Supporting information related to Experimental Procedure (DOCX 36 KB)

## Data Availability

Data are deposited in the Sequence Read Archive https://www.ncbi.nlm.nih.gov/bioproject/PRJNA787911.
