# Supplementary material for: Scale dependency of ectomycorrhizal fungal community assembly processes in Mediterranean mixed forests
Source: Mycorrhiza. 2022 Jun 4;32(3-4):315–25. doi: 10.1007/s00572-022-01083-4 (PMC9184349; doi:10.1007/s00572-022-01083-4)
Supplement: Supplementary file 1 — Appendix 1. Supporting information with figures and tables (DOCX 118 KB) [file 572_2022_1083_MOESM1_ESM.docx]

**Scale-dependency of ectomycorrhizal fungal community assembly processes in Mediterranean mixed forests**

**Mycorrhiza**

Prieto-Rubio J^1,2,3*^, Garrido JL^1,4^, Pérez-Izquierdo L^2,5^, Alcántara JM^6,7^, Azcón-Aguilar C^1^, López-García A^1,6,7†^, Rincón A^2†^

^1^Department of Soil Microbiology and Symbiotic Systems, Estación Experimental del Zaidín (EEZ), CSIC, Granada, Spain

^2^Department of Soil, Plant and Environmental Quality, Instituto de Ciencias Agrarias (ICA), CSIC, Madrid, Spain

^3^Escuela Internacional de Doctorado, Universidad Rey Juan Carlos (URJC), Madrid, Spain

^4^Department of Evolutionary Ecology, Estación Biológica de Doñana (EBD), CSIC, Seville, Spain

^5^BC3 Basque Centre For Climate Change, Scientific Campus of the University of the Basque Country, Leioa, Spain

^6^Department of Animal Biology, Plant Biology and Ecology, Universidad de Jaén, Jaén, Spain

^7^Instituto Interuniversitario de Investigación del Sistema Tierra en Andalucía (IISTA), Granada, Spain

***Corresponding author:** Jorge Prieto-Rubio; e-mail: jorge.prieto@eez.csic.es

Estación Experimental del Zaidín, Consejo Superior de Investigaciones Científicas (CSIC), 1, Rd. Profesor Albareda, 18008, Granada, Spain

***Appendix 1. Supplementary Tables and Figures***


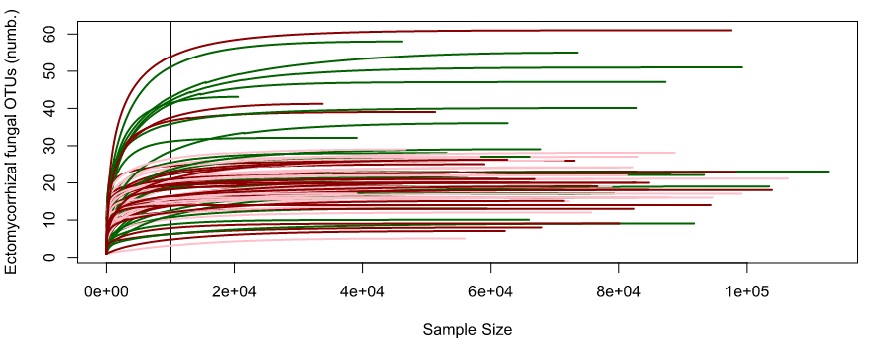


**Figure S1**. Rarefaction curves referred to abundance data of ectomycorrhizal fungal OTUs in each sample, showing those corresponding to *Cistus albidus* (green), *Quercus faginea* (pink) and *Quercus ilex* (dark red).

**Table S1**. Soil abiotic variables (A) and ectomycorrhizal fungal diversity indices (B) by host plant and season at each site (mean ± standard error). Means not sharing a letter differ significantly according to Tukey HSD test (p < 0.05). OM: organic matter; GM: gravimetric moisture. S: fungal richness; NTI: nearest taxon index.

| **(A) Site** | **Host plant** | **pH** | **OM (%)** | | **GM (%)** | | | |
| --- | --- | --- | --- | --- | --- | --- | --- | --- |
| Jaén | *Cistus albidus* | 7.7 ± 0.1 a | 13.7 ± 1.0 abc | | 21.1 ± 2.0 a | | | |
|  | *Quercus faginea* | 7.7 ± 0.1 a | 16.7 ± 2.6 ab | | 21.6 ± 1.9 a | | | |
|  | *Quercus ilex* | 7.7 ± 0.1 a | 18.9 ± 2.8 a | | 24.4 ± 1.7 a | | | |
| Segura | *Cistus albidus* | 7.1 ± 0.2 b | 9.1 ± 1.2 c | | 20.8 ± 1.3 a | | | |
|  | *Quercus faginea* | 6.4 ± 0.1 c | 7.9 ± 0.7 c | | 20.5 ± 0.9 a | | | |
|  | *Quercus ilex* | 6.3 ± 0.2 c | 9.6 ± 1.0 c | | 20.7 ± 1.4 a | | | |
|  | | | | | | | |  |
| **Site** | **Season** | **pH** | | **OM (%)** | | **GM (%)** | | |
| Jaén | Autumn | 7.9 ± 0.1 a | | 15.4 ± 1.5 a | | 27.7 ± 0.8 a | | |
|  | Spring | 7.5 ± 0.0 b | | 17.7 ± 2.4 a | | 16.9 ± 1.2 c | | |
| Segura | Autumn | 6.8 ± 0.2 c | | 3.5 ± 0.7 b | | 21.9 ± 0.8 b | | |
|  | Spring | 6.3 ± 0.1 d | | 9.5 ± 0.9 b | | 19.2 ± 1.1 bc | | |
|  |  |  | |  | |  | |  |
| **(B) Site** | **Host plant** | **S_Total_** | | **S_Ascomycetes_** | **S_Basidiomycetes_** | | **NTI_Regional_** | |
| Jaén | *Cistus albidus* | 21.4 ± 1.7 b | | 6.2 ± 0.8 a | 15.2 ± 1.2 b | | 0.6 ± 0.1 a | |
|  | *Quercus faginea* | 20.7 ± 1.2 b | | 4.6 ± 0.5 ab | 16.1 ± 1.2 b | | 0.6 ± 0.1 a | |
|  | *Quercus ilex* | 21.1 ± 1.2 b | | 3.8 ± 0.1 ab | 17.3 ± 1.4 b | | 0.6 ± 0.1 a | |
| Segura | *Cistus albidus* | 37.2 ± 4.3 a | | 6.1 ± 0.8 a | 31.0 ± 4.1 a | | 0.6 ± 0.1 a | |
|  | *Quercus faginea* | 26.9 ± 3.9 ab | | 3.1 ± 0.5 b | 23.7 ± 3.6 ab | | 0.4 ± 0.1 a | |
|  | *Quercus ilex* | 22.6 ± 1.8 b | | 2.6 ± 0.5 b | 20.0 ± 1.6 b | | 0.2 ± 0.2 a | |
|  |  |  | |  |  | | |  |
| **Site** | **Season** | **S_Total_** | | **S_Ascomycetes_** | **S_Basidiomycetes_** | | | **NTI_Regional_** |
| Jaén | Autumn | 21.0 ± 1.3 b | | 5.4 ± 0.6 a | 15.5 ± 1.1 b | | | 0.7 ± 0.1 a |
|  | Spring | 21.1 ± 0.8 b | | 4.2 ± 0.4 a | 16.9 ± 0.8 b | | | 0.5 ± 0.1 ab |
| Segura | Autumn | 25.5 ± 2.4 ab | | 4.0 ± 0.6 a | 21.5 ± 2.0 ab | | | 0.5 ± 0.1 ab |
|  | Spring | 32.0 ± 3.6 a | | 3.7 ± 0.5 a | 28.3 ± 3.3 a | | | 0.2 ± 0.1 b |

**Table S2**. Variations of ectomycorrhizal fungal community Nearest Taxon Index (NTI) at different spatial scales (regional, local, plot, host plant) compared with null expectations (*t* test). Mean NTI values with their respective confidence intervals (CI) at 95% are shown at each habitat scale. Asterisks on t value for a given site and habitat scale denote significant differences between observed and null communities: ‘***’ p < 0.001. No variation across scales was detected (tested via Tukey’ test).

| **ECM fungal phylodiversity** | ***t*** | **Mean** | **CI (95%)** |
| --- | --- | --- | --- |
| NTI_Regional_ | 8.06 *** | 0.48 | [0.35 , 0.58] |
| NTI_Local_ | 7.06 *** | 0.44 | [0.32 , 0.57] |
| NTI_Plot_ | 6.20 *** | 0.40 | [0.27 , 0.53] |
| NTI_Plant_ | 5.84 *** | 0.39 | [0.26 , 0.52] |

| **Variable** | **PCA1** | **PCA2** | **PCA3** | **PCA4** | **PCA5** | **PCA6** | **PCA7** | **PCA8** | **PCA9** | **PCA10** | **PCA11** |
| --- | --- | --- | --- | --- | --- | --- | --- | --- | --- | --- | --- |
| **pH** | -0.023 | 0.006 | -0.019 | -0.116 | **0.987** | 0.000 | 0.001 | -0.010 | -0.008 | 0.035 | 0.100 |
| **OM** | **-0.900** | -0.434 | -0.015 | -0.023 | -0.022 | 0.000 | 0.000 | 0.002 | 0.002 | -0.003 | 0.004 |
| **GM** | -0.433 | **0.900** | -0.031 | -0.011 | -0.017 | 0.000 | 0.000 | -0.002 | 0.000 | -0.007 | -0.001 |
| **PCOA1** | -0.027 | -0.001 | -0.035 | **0.992** | 0.115 | 0.000 | 0.001 | -0.004 | 0.013 | 0.014 | 0.001 |
| **PCOA2** | 0.029 | -0.021 | **-0.999** | -0.032 | -0.022 | 0.000 | 0.003 | 0.004 | -0.001 | -0.004 | 0.001 |
| **PCNM1** | 0.005 | 0.003 | 0.003 | 0.005 | -0.095 | -0.064 | 0.135 | 0.021 | 0.302 | 0.107 | **0.930** |
| **PCNM2** | 0.001 | 0.001 | 0.005 | 0.006 | 0.021 | -0.264 | **0.548** | **0.733** | -0.112 | -0.280 | -0.044 |
| **PCNM3** | 0.004 | -0.003 | 0.002 | 0.001 | 0.031 | **0.533** | -0.043 | 0.009 | **0.424** | **-0.730** | -0.008 |
| **PCNM4** | 0.000 | 0.003 | 0.001 | 0.007 | -0.018 | 0.381 | **-0.593** | **0.599** | -0.303 | 0.141 | 0.179 |
| **PCNM5** | -0.002 | 0.001 | 0.001 | 0.000 | -0.017 | **0.698** | **0.572** | -0.099 | -0.278 | 0.313 | 0.020 |
| **PCNM6** | -0.002 | 0.004 | -0.002 | -0.017 | 0.019 | 0.099 | 0.025 | 0.306 | **0.739** | **0.508** | -0.300 |
| Variable type: | ENV | ENV | ENV | **ENV** | ENV | SPATIAL | SPATIAL | SPATIAL | **ENV** | SPATIAL | SPATIAL |
|  | MEASURED | MEASURED | MEASURED | **MEASURED** | MEASURED | SPATIAL | SPATIAL | SPATIAL | **UNMEASURED** | SPATIAL | SPATIAL |

**Table S3**. Loadings of Principal Component Analysis axes (PCA) in relation with explanatory variables used to calculate PCA: soil variables (OM = organic matter; GM = gravimetric moisture), phylogeny of plant species (decomposed via Principal Coordinates Analysis, PCOAs), and spatial variables (decomposed as Principal Coordinates of Neighbour Matrices, PCNMs). Variables that heavily loaded a PCA axis are marked in bold. PCA axes were classed as environmental measured variables (ENV MEASURED) when heavier loadings were associated with the measured soil variables and PCoAs; spatial variables when PCA axes were loaded by PNCM (SPATIAL); and spatially-structured environmental unmeasured variables in the cases when spatial variables significantly explained βNTI in the dbRDA (ENV UNMEASURED).

| **Fungal Family** | **PCoA 5** | **PCoA 9** | **PCoA 36** | **PCoA 44** | **PCoA 57** |
| --- | --- | --- | --- | --- | --- |
| Albatrellaceae | **-0.18 .** | -0.03 | -0.07 | 0.03 | 0.13 |
| Amanitaceae | 0.06 | -0.05 | -0.02 | -0.10 | -0.07 |
| Atheliaceae | 0.17 | -0.07 | -0.02 | 0.09 | -0.12 |
| Bankeraceae | 0.07 | -0.06 | 0.07 | 0.09 | -0.05 |
| Boletaceae | 0.13 | 0.08 | **-0.18 .** | -0.01 | 0.10 |
| Cantharellaceae | 0.01 | 0.05 | **-0.18 .** | -0.05 | -0.09 |
| Clavulinaceae | -0.08 | 0.09 | 0.08 | 0.12 | -0.06 |
| Cortinariaceae | -0.11 | -0.10 | -0.13 | 0.14 | **-0.17 .** |
| Elaphomycetaceae | -0.17 | -0.01 | -0.05 | 0.11 | -0.17 |
| Endogonaceae | -0.08 | 0.05 | -0.12 | 0.04 | -0.04 |
| Gloniaceae | -0.02 | -0.12 | -0.08 | 0.01 | 0.04 |
| **Gomphidiaceae** | **-0.23 *** | 0.07 | -0.08 | 0.08 | 0.07 |
| Helotiaceae | -0.11 | -0.15 | -0.16 | -0.04 | 0.04 |
| **Helvellaceae** | 0.14 | -0.10 | 0.15 | 0.14 | **0.26 *** |
| Hydnaceae | -0.02 | 0.13 | 0.01 | -0.03 | **-0.19 .** |
| Hydnangiaceae | -0.07 | -0.09 | -0.02 | 0.15 | 0.09 |
| Hygrophoraceae | 0.00 | **-0.18 .** | 0.07 | 0.12 | 0.01 |
| Hymenosgastraceae | -0.03 | 0.05 | -0.08 | 0.05 | -0.06 |
| Inocybaceae | -0.05 | 0.15 | -0.12 | 0.04 | -0.11 |
| Lyophyllaceae | 0.07 | -0.16 | 0.01 | **0.18 .** | -0.12 |
| Melanogastraceae | -0.08 | **0.20 .** | -0.05 | -0.01 | 0.07 |
| **Pezizaceae** | -0.01 | -0.07 | 0.07 | -0.13 | **0.25 *** |
| Pyronemataceae | 0.00 | -0.03 | 0.13 | 0.00 | 0.03 |
| Rhizopogonaceae | 0.02 | -0.01 | -0.17 | 0.00 | -0.03 |
| **Russulaceae** | **-0.28 **** | -0.11 | **-0.18 .** | -0.02 | -0.06 |
| Sebacinaceae | 0.17 | **0.18 .** | 0.07 | 0.02 | -0.07 |
| Serendipitaceae | 0.06 | -0.01 | 0.01 | 0.08 | 0.02 |
| Suillaceae | 0.03 | 0.02 | -0.03 | **-0.20 .** | 0.05 |
| Thelephoraceae | **-0.18 .** | 0.01 | 0.05 | 0.01 | 0.15 |
| Tricholomataceae | 0.04 | -0.01 | 0.10 | -0.02 | -0.04 |
| **Tuberaceae** | 0.04 | 0.02 | **0.22 *** | -0.02 | -0.02 |

**Table S4**. Correlations among ECM fungal community phylogenetic turnover (βNTI) with relative abundance of ECM fungal taxa at Family level. Phylogenetic turnover was described by calculating the PCoA axes. Only the axes significantly related with PCAs 4 (PCoAs 5, 9 and 36) and 9 (PCoAs 44 and 57) were used in correlations with fungal families. Significant Spearman correlations are marked in bold and with codes: ‘**’ < 0.01; ‘*’ < 0.05; ‘.’ < 0.1.
