# Supplementary material for: Scale dependency of ectomycorrhizal fungal community assembly processes in Mediterranean mixed forests
Source: Mycorrhiza. 2022 Jun 4;32(3-4):315–25. doi: 10.1007/s00572-022-01083-4 (PMC9184349; doi:10.1007/s00572-022-01083-4)
Supplement: Supplementary file 2 — Appendix 2. Supporting information related to Experimental Procedure (DOCX 36 KB) [file 572_2022_1083_MOESM2_ESM.docx]

**Scale-dependency of ectomycorrhizal fungal community assembly processes in Mediterranean mixed forests**

**Mycorrhiza**

Prieto-Rubio J^1,2,3*^, Garrido JL^1,4^, Pérez-Izquierdo L^2,5^, Alcántara JM^6,7^, Azcón-Aguilar C^1^, López-García A^1,6,7†^, Rincón A^2†^

^1^Department of Soil Microbiology and Symbiotic Systems, Estación Experimental del Zaidín (EEZ), CSIC, Granada, Spain

^2^Department of Soil, Plant and Environmental Quality, Instituto de Ciencias Agrarias (ICA), CSIC, Madrid, Spain

^3^Escuela Internacional de Doctorado, Universidad Rey Juan Carlos (URJC), Madrid, Spain

^4^Department of Evolutionary Ecology, Estación Biológica de Doñana (EBD), CSIC, Seville, Spain

^5^BC3 Basque Centre For Climate Change, Scientific Campus of the University of the Basque Country, Leioa, Spain

^6^Department of Animal Biology, Plant Biology and Ecology, Universidad de Jaén, Jaén, Spain

^7^Instituto Interuniversitario de Investigación del Sistema Tierra en Andalucía (IISTA), Granada, Spain

***Corresponding author:** Jorge Prieto-Rubio; e-mail: jorge.prieto@eez.csic.es

Estación Experimental del Zaidín, Consejo Superior de Investigaciones Científicas (CSIC), 1, Rd. Profesor Albareda, 18008, Granada, Spain

***Appendix 2. Supplementary Methods***

*Sampling design*

To study the ECM fungal communities associated with Jaén and Segura forests, we selected three representative ECM plant species (*Cistus albidus* L., *Q. faginea* and *Q. ilex*). The study was conducted in four different forest stands in each site, separated by a maximum of 5 km from each other. In each stand, one 50 x 50 m plot was established, as previously described in Alcántara et al. (2018). To capture part of the temporal variability of ECM fungal communities, sampling was carried out in autumn 2016 and spring 2017. Four plants per species were randomly selected in each plot (2 individuals per season) and sampled with a minimum distance of 10 m to avoid re-sampling the same ECM fungal community. This yielded n = 32 individuals for *Q. ilex*, and n = 31 and n = 29 respectively for *Q. faginea* and *C. albidus*, for which a few less adult individuals distinct to those sampled in autumn were found in spring. The total number of plant individuals sampled was n = 92.

*Ectomycorrhizal sampling*

After removing the litter layer, roots were excavated in 10 x 10 x 20 cm holes at approximately 0.5 m from the trunk in N, SE and SW directions traced back to the target plant, and kept at 4 ºC until processing. Once in the lab, the three subsamples were pooled into a single composite sample per plant individual. Roots were separated from soils, coarse roots (diameter > 2 mm) kept apart, and the remaining roots gently washed with tap water over 2 and 0.5 mm sieves for collecting root tips. All ECM root tips were carefully sorted per sample under a Carl Zeiss Stemi 2000 stereomicroscope (Rincón et al. 2014), and stored at -20 ºC for further molecular analyses. The remaining soil was homogenized, air-dried and sieved (2 mm) for chemical analyses. Gravimetric soil moisture (GM) was determined as the difference in soil weight before and after oven-dried at 105 °C for 48 h. Soil pH was measured in soil solutions (1:5, w:v in H_2_O) and the organic matter (OM) determined by loss on ignition at 400 ºC for 4 h (Walkley and Black, 1934).

*Molecular analyses*

Ectomycorrhizas of 92 plant individuals were obtained. ECM root-tips (~ 30 mg per sample) were mixed with a pinch of polyvinylpyrrolidone (PVPP), and the DNA extracted with the Invisorb®DNA Plant HTS 96 Kit/C kit (Invitek GmbH, Berlin, Germany). The internal transcribed spacer region ITS-1 of the nuclear ribosomal DNA was amplified with the primer pair ITS1F-ITS2 (Gardes and Bruns 1993) in three independent 20 μl PCR reactions. Each sample contained 2 μl of 10 x polymerase buffer, 2.4 μl of 25 mM MgCl_2_, 1.12 μl of 10 mg ml^-1^ BSA, 0.4 μl of 10 mM nucleotide mix, 0.4 μl of 10 mM forward/reverse primers (adaptors A-tag-ITS1F/B-ITS2), and 0.2 μl of AmpliTaqGold polymerase (5 U μl^-1^) (Applied Biosystems, Carlsbad, CA, USA). Negative controls without DNA using purified water (Sigma Aldrich, Inc.) were included in all runs to detect possible contaminations. DNA amplifications were carried out by using the protocol described in Pérez-Izquierdo et al. (2020) under the following PCR conditions: 3 min at 94°C, 30 cycles of 1 min at 94°C, 30 s at 53°C and 45 s at 72°C, and a final step of 10 min at 72°C. Independent reactions were combined per sample, and PCR products were purified (AMPure magnetic beads, Beckman Coulter Inc., CA, USA), quantified (PicoGreen bioanalyzer, LifeTechonologies, Carlsbad, CA, USA) and pooled to build two equimolar libraries containing 46 uniquely tagged samples (autumn and spring). Libraries of amplicons were sequenced by Illumina-MiSeq high-throughput sequencing (2 x 300 bp paired end reads) in an external service (Fundación Parque Científico de Madrid, Spain).

*Bioinformatics*

The sequences were processed with the R open-source DADA2 pipeline v1.16 (Callahan et al. 2016; R Core Team 2021), with default parameters, by performing the filtering, de-replication, chimera identification and merging paired-end reads. The workflow generated an abundance matrix in which the number of sequences (hereafter reads) obtained for each amplicon sequence variant (ASV) in the samples was used as proxy of abundance. Fungal taxonomic categories were then assigned to ASVs using the RDP algorithm and the UNITE database v7.2 (Abarenkov et al. 2010; Cole et al*.* 2014). A clustering at 97% similarity was carried out by implementing vsearch (Rognes et al. 2016) in mothur (Schloss et al. 2009) to get Operational Taxonomic Units (OTUs). The OTUs were further curated by applying the LULU algorithm to enhance biodiversity metrics (Frøslev et al. 2017). The database was normalized by dividing the number of sequences per OTU in a sample by the total number of sequences of that sample.

The taxonomic assignment was used to classify OTUs by fungal guild (i.e., ECM fungi, arbuscular mycorrhizal fungi, pathogens, saprotrophs, etc.) with the FUNGuild database v1.0 (Nguyen et al*.* 2016) and bibliographic support (Agerer 2006; Tedersoo and Smith 2013; Tedersoo et al. 2014). From the final output of 983 fungal OTUs and 6,847,682 reads, a subset of 449 OTUs and 6,582,941 reads were associated with the symbiotic ECM lifestyle and was used as ECM fungal abundance matrix. Homogeneous sequencing depth across samples was confirmed by rarefaction analysis (*vegan* R package, Oksanen et al. 2019).

**References**

Abarenkov K, Henrik Nilsson R, Larsson KH et al (2010) The UNITE database for molecular identification of fungi–recent updates and future perspectives. New Phytol 186:281-285. http://doi: 10.1111/j.1469-8137.2009.03160.x

Agerer R (2006) Fungal relationships and structural identity of their ectomycorrhizae. Mycol Prog 5:67-107. http://doi: 10.1007/s11557-006-0505-x

Alcántara JM, Pulgar M, Trøjelsgaard K et al (2018) Stochastic and deterministic effects on interactions between canopy and recruiting species in forest communities. Funct Ecol 32:2264–2274. http://doi: 10.1111/1365-2435.13140

Callahan BJ, McMurdie PJ, Rosen MJ et al (2016) DADA2: High-resolution sample inference from Illumina amplicon data. Nat Methods 13:581–583. http://doi: 10.1038/nmeth.3869

Cole JR, Wang Q, Fish JA et al (2014) Ribosomal Database Project: Data and tools for high throughput rRNA analysis. Nucleic Acids Res 42:633–642. http://doi: 10.1093/nar/gkt1244

Frøslev TG, Kjøller R, Bruun HH et al (2017) Algorithm for post-clustering curation of DNA amplicon data yields reliable biodiversity estimates. Nat Commun 8:1-11. http://doi: 10.1038/s41467-017-01312-x

Gardes M, Bruns TD (1993) ITS primers with enhanced specificity for basidiomycetes‐application to the identification of mycorrhizae and rusts. Mol Ecol 2:113-118. https://doi.org/10.1111/j.1365-294X.1993.tb00005.x

Nguyen NH, Song Z, Bates ST et al (2016) FUNGuild: An open annotation tool for parsing fungal community datasets by ecological guild. Fungal Ecol 20:241–248. http://doi: 10.1111/j.1365-294X.1993.tb00005.x

Oksanen J, Blanchet FG, Friendly M et al (2019) vegan: Community Ecology Package. R package version 2.5-4. <https://CRAN.R-project.org/package=vegan>

Pérez-Izquierdo L, Zabal-Aguirre M, Verdú M et al (2020) Ectomycorrhizal fungal diversity decreases in Mediterranean pine forests adapted to recurrent fires. Mol Ecol 29:1–14. http://doi: 10.1111/mec.15493

R Core Team (2021) R: A language and environment for statistical computing. R Foundation for Statistical Computing, Vienna, Austria. URL https://www.R-project.org/.

Rincón A, Santamaría BP, Ocaña L, Verdú M (2014) Structure and phylogenetic diversity of post-fire ectomycorrhizal communities of maritime pine. Mycorrhiza, 24:131–141. http://doi: 10.1007/s00572-013-0520-0

Rognes T, Flouri T, Nichols B et al (2016) VSEARCH: A versatile open source tool for metagenomics. PeerJ 4:e2584. http://doi: 10.7717/peerj.2584

Schloss PD, Westcott SL, Ryabin T et al (2009) Introducing mothur: Open-source, platform-independent, community-supported software for describing and comparing microbial communities. *Appl Environ Microbiol* *75*:7537–7541. https://doi.org/10.1128/AEM.01541-09

Tedersoo L, Smith ME (2013) Lineages of ectomycorrhizal fungi revisited: Foraging strategies and novel lineages revealed by sequences from belowground. Fungal Biol Rev 27:83–99. http://doi: 10.1128/AEM.01541-09

Tedersoo L, Bahram M, Ryberg M et al (2014) Global biogeography of the ectomycorrhizal/sebacina lineage (Fungi, Sebacinales) as revealed from comparative phylogenetic analyses. Mol Ecol 23:4168-4183. http://doi: 10.1111/mec.12849

Walkley A, Black IA (1934) An examination of the Degtjareff method for determining soil organic matter, and a proposed modification of the chromic acid titration method. Soil science 37:29-38.
